# Delaying Effects of Prolactin and Growth Hormone on Aging Processes in Bovine Oocytes Matured In Vitro

**DOI:** 10.3390/ph14070684

**Published:** 2021-07-16

**Authors:** Galina N. Singina, Ekaterina N. Shedova, Alexander V. Lopukhov, Olga S. Mityashova, Irina Y. Lebedeva

**Affiliations:** Department of Animal Biotechnology and Molecular Diagnostics, L.K. Ernst Federal Research Center for Animal Husbandry, 142132 Podolsk, Russia; g_singina@mail.ru (G.N.S.); shedvek@yandex.ru (E.N.S.); vubi_myaso@mail.ru (A.V.L.); mityashova_o@mail.ru (O.S.M.)

**Keywords:** prolactin, growth hormone, oocyte aging, spontaneous parthenogenetic activation, apoptosis, developmental capacity, receptors, cumulus cells, signaling pathways

## Abstract

Aging processes accelerate dramatically in oocytes that have reached the metaphase-II (M-II) stage. The present work aimed to study the patterns and intracellular pathways of actions of prolactin (PRL) and growth hormone (GH) on age-associated changes in bovine M-II oocytes aging in vitro. To this end, we analyzed spontaneous parthenogenetic activation (cytogenetic assay), apoptosis (TUNEL assay), and the developmental capacity (IVF/IVC) of in vitro-matured oocytes after prolonged culturing. Both PRL and GH reduced the activation rate of aging cumulus-enclosed oocytes (CEOs) and denuded oocytes (DOs), and their respective hormone receptors were revealed in the ova. The inhibitor of Src-family tyrosine kinases PP2 eliminated the effects of PRL and GH on meiotic arrest in DOs, whereas the MEK inhibitor U0126 only abolished the PRL effect. Furthermore, PRL was able to maintain the apoptosis resistance and developmental competence of aging CEOs. The protein kinase C inhibitor calphostin C suppressed both the actions of PRL. Thus, PRL and GH can directly support meiotic arrest in aging M-II oocytes by activating MAP kinases and/or Src-family kinases. The effect of PRL in maintaining the developmental capacity of aging oocytes is cumulus-dependent and related to the pro-survival action of the protein kinase C-mediated signal pathway.

## 1. Introduction

The developmental competence of mammalian oocytes depends on their quality. Once the oocyte reaches the metaphase-II (M-II) stage, both in vivo and in vitro, it undergoes a time-dependent process of deterioration named postovulatory oocyte aging [1,2]. Postovulatory aging triggers different morphological and functional changes in oocytes, primarily spontaneous parthenogenetic activation (SPA), apoptosis, and abnormal transformations of organelles [2,3]. As a consequence, when fertilization or the artificial activation of the matured ovum fail to occur at the appropriate time, its quality is compromised, leading to impaired embryo development and offspring health [2,4,5]. The rapid loss of M-II oocyte fitness is one of the factors limiting the efficiency of assisted reproductive technologies (ART) in various species, including humans [6,7]. In addition, the spontaneous activation and apoptosis associated with ovum aging are believed to be contributing to the decline in the population of some threatened mammalian species [8]. 

Increasing the knowledge of mechanisms and factors that regulate the senescence of mature oocytes can help to expand the window for fertilization, and thereby enhance reproduction outcomes. According to current notions, the aging process of mammalian ova is underlaid by various molecular and biochemical alterations that cause deleterious morphological and functional changes [1,2,3]. Such alterations involve the enhanced generation of reactive oxygen species (ROS), reduced intracellular levels of adenosine triphosphate (ATP) and associated adenosine 3′,5′-cyclic monophosphate (cAMP), abnormal Ca^2+^ oscillations, the destabilization of the maturation-promoting factor (MPF), decreased expressions of antiapoptotic factors, increased expressions of proapoptotic factors, the activation of caspase-3, and improper epigenetic modifications [9,10,11,12,13]. In addition, different detrimental changes in kinase signaling pathways, including mitogen-activated protein kinase (MAPK), protein kinase C (PKC), Akt, and Src-family tyrosine kinases (SFKs), have been demonstrated in aging M-II oocytes [10,14,15,16].

The successful control of the female fertility requires information regarding the physiological factors, especially the endocrine ones, responsible for the M-II oocyte defense against precocious senescence. Oxidative stress, leading to mitochondrial dysfunction, disturbances in calcium homeostasis and subsequent oocyte SPA or apoptosis, is considered the main factor inducing postovulatory aging [2,3]. Concurrently, the aging-accelerating role of cumulus cells surrounding mature ova has been well established, suggesting the participation of mechanisms other than oxidative stress [17,18]. The hormone melatonin, secreted by the pineal gland, has been shown to be able to protect mammalian oocytes matured in vivo and in vitro from time-dependent oxidative stress, and thereby inhibit the age-related deterioration of their quality [19,20]. However, relatively little is known about the pathways of action of other endocrine factors on the aging of M-II oocytes.

Postovulatory aging is usually studied in animal models, mainly in laboratory rodents, because of the ethics, costs, and juristic limitations associated with using human oocytes. Meanwhile, the bovine model may be preferable, due to the similarities of meiosis regulation mechanisms and early embryo development here with those in humans [21,22,23]. In cattle, in vitro aging results in the same functional and molecular changes in oocytes as in other species, adversely affecting ART outcomes [24,25]. We have previously found the similar decelerating effects of two related hormones, prolactin (PRL) and growth hormone (GH), on age-associated alterations in M-II chromosomes during the prolonged culture of bovine oocytes matured in vitro. Concurrently, these effects are mediated by cumulus cells and related to the modulation of the activity of Src-family tyrosine kinases, Akt, protein kinase C, and NO-synthase isoforms [26,27]. Thus, the present study was performed to test the hypothesis that PRL and GH are also able to suppress other aging-associated functional changes in bovine M-II oocytes through the above signaling pathways. To this end, we analyzed SPA, apoptosis, and the developmental capacity of the in vitro-matured oocytes after prolonged culture with and without PRL and GH. Our findings indicate for the first time that both hormones exert a direct maintaining action on meiotic arrest at the M-II stage in aging bovine ova. At the same time, only PRL can inhibit cumulus-mediated apoptotic processes in the mature oocytes, and thereby support their competence for further embryonic development. The revealed differences in the effects of the related hormones are obviously attributed to the PRL activation of some signal cascades, unaffected by GH, in bovine oocytes that protect these latter from the adverse influence of senescent cumulus cells. 

## 2. Results

In all our experiments, PRL (50 ng/mL) and GH (10 ng/mL) were used at the concentrations at which they had an inhibitory effect on the destructive changes in M-II chromosomes in aging bovine cumulus-enclosed oocytes (CEOs) [26]. The applied concentrations of different signaling inhibitors were the same as, or very close to, those that were effective in suppressing PRL and GH’s actions on bovine oocytes or other mammalian cells [26,27,28,29].

### 2.1. Effects of PRL and GH on Spontaneous Parthenogenetic Activation (SPA) of Aging Bovine Oocytes

**Figure 1:**
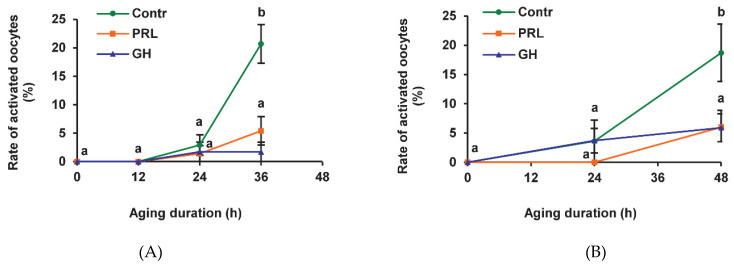
Effects of prolactin (PRL, 50 ng/mL) and growth hormone (GH, 10 ng/mL) on spontaneous parthenogenetic activation during the aging of bovine cumulus-enclosed oocytes (CEOs) (**A**) and denuded oocytes (DOs) (**B**). Data represent means ± SEM of 4–5 replicates using 71–104 oocytes per treatment. Means marked with different letters differ significantly (at least *p* < 0.05).

We have previously shown that the frequency of SPA reaches a maximum after 36 h of prolonged culturing of bovine CEOs, whereas it continues to increase between 36 h and 48 h in the case of denuded oocytes (DOs) [24]. In the present research, most of the CEOs and DOs maintained meiotic arrest at the M-II stage up to 24 h in the aging medium (Figure 1). After 36 h and 48 h of aging, the rates of activated CEOs and DOs, respectively, were similarly increased to 18.7–21.1% (*p* < 0.001). Representative images of the cytogenetic preparations, illustrating the morphology of the nuclear material at various stages of SPA in aged bovine ova, are shown in Figure S1. The addition of PRL or GH to the aging medium resulted in a more than three-fold reduction (*p* < 0.001) in the SPA frequency of both CEOs (Figure 1A) and DOs (Figure 1B), suggesting the direct effect of the hormones on bovine oocytes.

**Figure 2:**
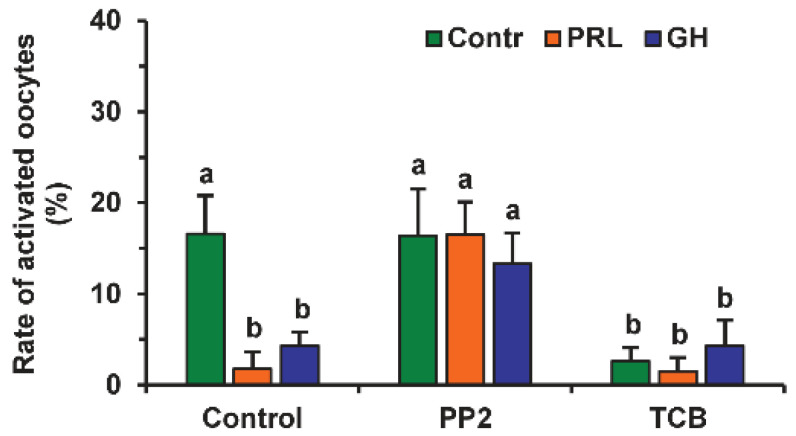
Effects of PRL (50 ng/mL) and GH (10 ng/mL) on spontaneous parthenogenetic activation during the 44 h aging of bovine DOs in the presence and in the absence of PP2 (20 μM), the inhibitor of Src-family tyrosine kinases, and triciribine (TCB, 50 μM), the inhibitor of Akt kinase. Data derived from 4 independent replicates (a total of 79–86 oocytes per each treatment group) are expressed as means ± SEM. Different letters indicate significant differences between means (at least *p* < 0.05).

The involvement of different signaling pathways in PRL and GH’s effects on the SPA of aging DOs was studied by testing the effects of the respective inhibitors. The signaling pathways, associated with SFKs, Akt and MAP kinases, and NO, were selected due to their role in meiosis regulation in mammals [7,30,31,32]. The inhibitor of SFKs 4-Amino-3-(4-chlorophenyl)-1-(t-butyl)-1*H*-pyrazolo[3,4-d]pyrimidine, 4-Amino-5-(4-chlorophenyl)-7-(t-butyl)pyrazolo[3,4-d]pyrimidine (PP2, 20 µM) eliminated the supporting effects of PRL and GH on meiotic arrest at the M-II stage, enhancing the rates of activated oocytes, respectively, from 1.8% to 16.5% and from 4.3% to 13.3% (at least *p* < 0.05), but it did not affect this rate in the control medium (Figure 2). Similar to PRL and GH, the inhibitor of Akt kinase triciribine (50 µM) decreased the frequency of oocyte SPA in the control medium (*p* < 0.001) several-fold, without changing the effects of the hormones themselves.

**Figure 3:**
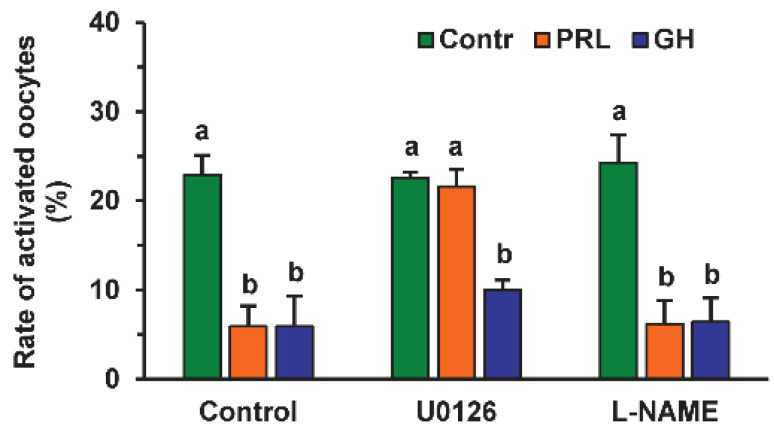
Effects of PRL (50 ng/mL) and GH (10 ng/mL) on spontaneous parthenogenetic activation during the 44 h aging of bovine DOs in the presence and in the absence of U0126 (20 μM), the MEK 1/2 inhibitor, and L-NAME (20 μM), the efficient eNOS and nNOS inhibitor (20 μM). Data derived from 4 independent replicates (a total of 71–80 oocytes per each treatment group) are expressed as means ± SEM. Different letters indicate significant differences between means (at least *p* < 0.05).

The MEK 1/2 (MAP/ERK kinase 1/2) inhibitor U0126 (20 µM) increased the rate of activated oocytes 3.7-fold during their prolonged culture in the medium with PRL (*p* < 0.01), but not in that with GH or without either hormone (Figure 3). At the same time, Nω-Nitro-L-arginine methyl ester hydrochloride (L-NAME, 20 μM), an efficient inhibitor of endothelial NO-synthase (eNOS) and neuronal NO-synthase (nNOS), had no effect on the frequency of oocyte SPA regardless of the presence of PRL or GH in the aging medium.

### 2.2. Localization of PRL and GH Receptors in Matured Bovine Oocytes

**Figure 4:**
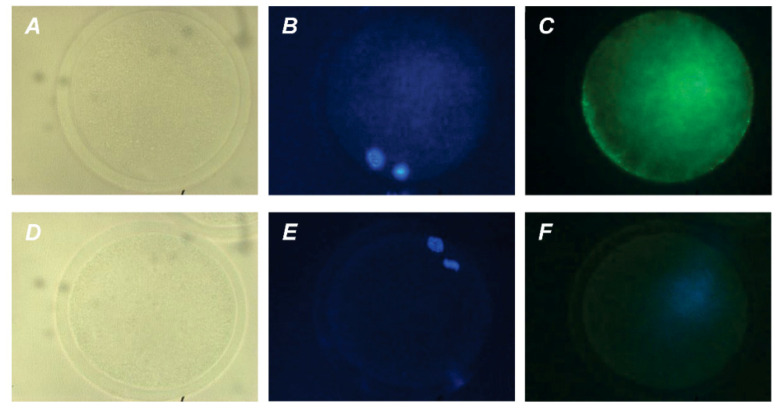
Immunofluorescent detection of PRL receptors in M-II oocytes after the 20 h maturation of bovine CEOs. (**A**,**D**) Bright-field images. (**B**,**E**) DNA staining with DAPI (blue). (**C**) Positive staining using the MA1-610 antibody and goat anti-mouse IgG conjugated with Alexa Fluor 488 (green). (**F**) Negative control performed by omitting the primary antibody. Original magnification: ×400.

The direct action of PRL and GH on bovine M-II oocytes was confirmed by the presence of their respective receptors in the matured ova. The expression of PRL receptors in the oocytes was revealed by both immunofluorescence (Figure 4) and immunocytochemistry (Figure S2), with the use of the MA1 610 antibody, which detects long and short PRL receptor isoforms in various ovarian cells, such as bovine cumulus cells and immature oocytes [26,33].

**Figure 5:**
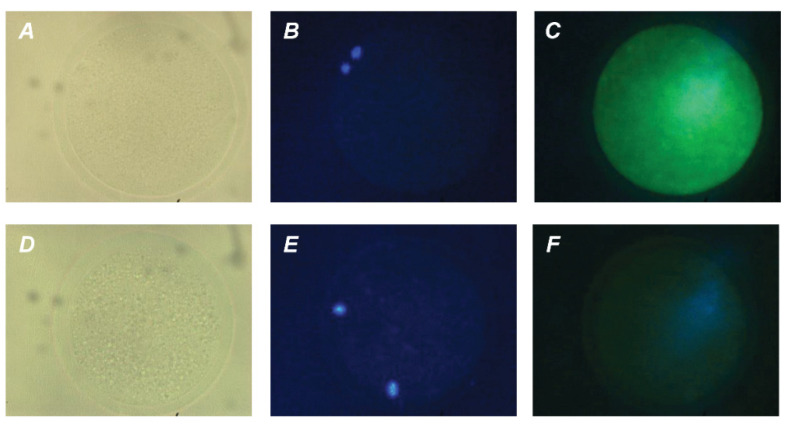
Immunofluorescent detection of GH receptors in M-II oocytes after the 20 h maturation of bovine CEOs. (**A**,**D**) Bright-field images. (**B**,**E**) DNA staining with DAPI (blue). (**C**) Positive staining using MAB 263 antibody and goat anti-mouse IgG conjugated with Alexa Fluor 488 (green). (**F**) Negative control performed by omitting the primary antibody. Original magnification: ×400.

Furthermore, in vitro-matured oocytes showed intense green staining (immunofluorescence; Figure 5) or brown staining (immunocytochemistry; Figure S2) with the anti-GH receptor antibody MAB 263. The antibody used has previously been extensively validated for immunochemical studies of GH receptors in various types of bovine tissues, including ovarian ones [26,34]. No specific immunoreactivity was found in negative controls performed by omitting the primary antibodies.

### 2.3. Effects of PRL on the Apoptosis of Aging Bovine Oocytes

**Figure 6:**
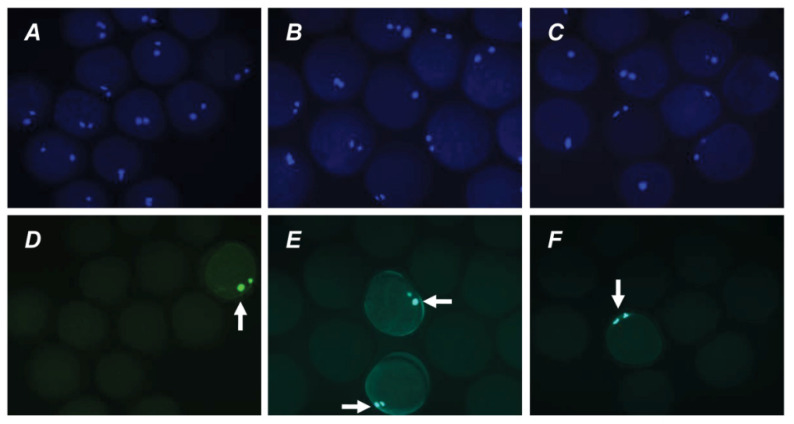
Representative images of apoptosis in matured or aged bovine CEOs. (**A**,**D**) Matured oocytes prior to aging. (**B**,**E**) Oocytes aged in the control medium. (**C**,**F**) Oocytes aged in the presence of PRL. (**A**–**C**) DAPI staining (blue). *(***D**–**F**) TUNEL staining (green). TUNEL-positive chromosomes are indicated by white arrows. Original magnification: ×200.

**Figure 7:**
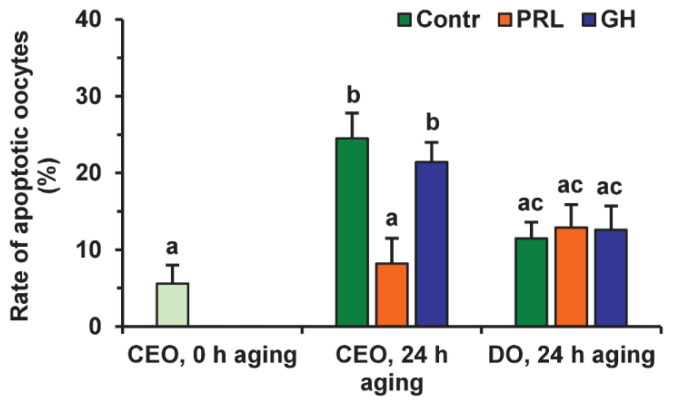
Effects of PRL (50 ng/mL) and GH (10 ng/mL) on oocyte apoptosis during the 24 h aging of bovine CEOs and DOs. Data represent means ± SEM of 4 replicates using 75–88 oocytes per treatment. Means marked with different letters differ significantly (at least *p* < 0.05).

Representative images illustrating the detection of bovine M-II oocytes at the late stages of apoptosis, provided by the Terminal Deoxynucleotidyl Transferase-Mediated 2′-Deoxyuridine 5′-Triphosphate (dUTP) Nick-End Labeling (TUNEL) assay, are shown in Figure S3. During 24 h of aging in the control medium, the rate of apoptotic CEOs increased from 5.6% (0 h) to 24.5% (24 h, *p* < 0.01), whereas the rate of apoptotic DOs rose insignificantly (to 11.5%) (Figure 6 and Figure 7).

After the prolonged culture with PRL, the rate of apoptotic CEOs was reduced as compared to the control group (up to 8.2%, *p* < 0.01), and did not differ from that prior to aging. At the same time, the impact of PRL on the apoptosis of aging oocytes disappeared when removing cumulus cells (Figure 7), which indicates the involvement of these latter in the anti-apoptotic action of the hormone. Meanwhile, no effect of GH on the frequency of oocyte apoptosis was observed in the case of either CEOs or DOs.

**Figure 8:**
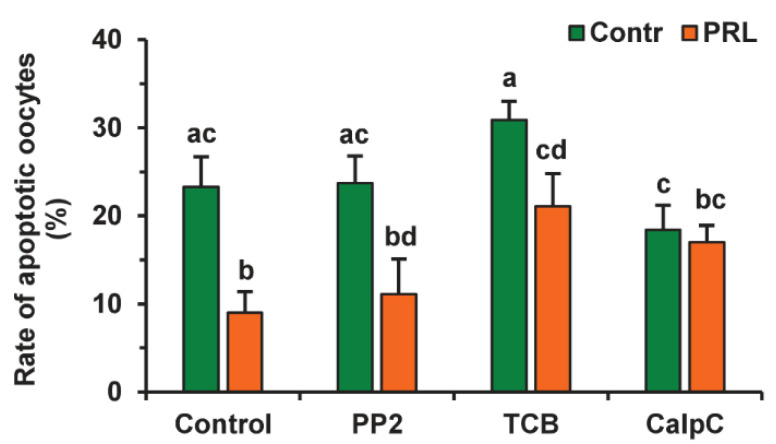
Effects of PRL (50 ng/mL) on oocyte apoptosis during the 24 h aging of bovine CEOs in the presence and in the absence of PP2 (20 μM), triciribine (TCB, 50 μM), and calphostin C (CalpC; 1 µM). Data represent means ± SEM of 4 replicates using 52–80 oocytes per treatment. Means marked with different letters differ significantly (at least *p* < 0.05).

To evaluate the participation of signaling pathways in the pro-survival effect of PRL on aging CEOs, we used the same inhibitors that suppressed the cumulus-mediated decelerating effect of PRL on age-associated alterations in M-II chromosomes in bovine ova [26]. The suppressive effect of PRL on oocyte apoptosis was found to be abolished by calphostin C (1 μM), a protein kinase C inhibitor, but not by triciribine (50 µM) or PP2 (20 µM) (Figure 8). Furthermore, triciribine increased 2.3-fold (*p* < 0.05) the apoptosis frequency of CEOs, aging in the presence of PRL, while the frequency was unaffected by calphostin C or PP2.

### 2.4. Effects of PRL on the Developmental Capacity of Aging Bovine Oocytes

**Figure 9:**
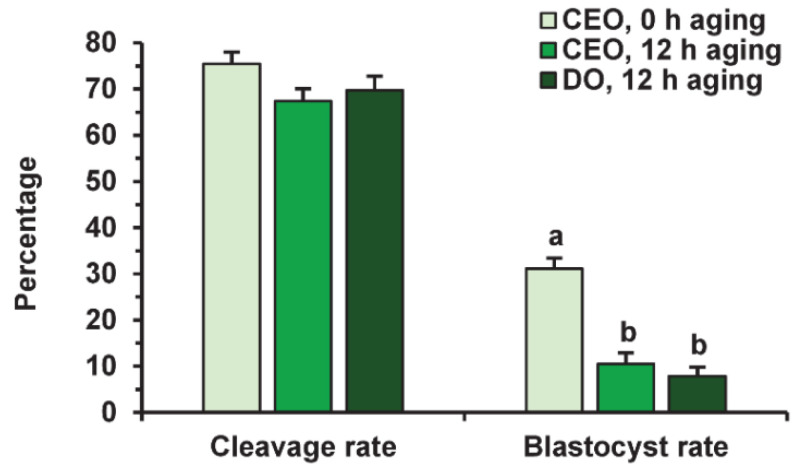
The developmental capacity of in vitro matured and aged bovine CEOs and DOs. Data represent means ± SEM of 6 replicates using 153–172 oocytes per treatment. Means marked with different letters differ significantly (*p* < 0.001).

**Figure 10:**
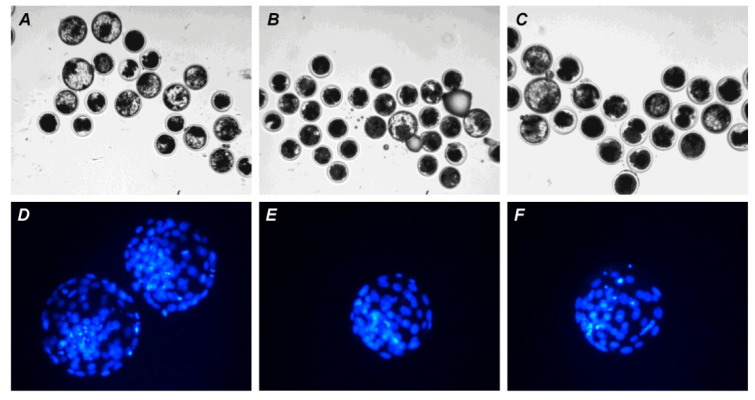
Representative images of embryos (day 7) derived from in vitro-matured or aged bovine CEOs. (**A**,**D**) Oocytes were fertilized immediately after IVM. (**B**,**E**) Oocytes were fertilized following 12 h of aging in the control medium. (**C**,**F**) Oocytes were fertilized following 12 h of aging in the medium containing PRL. (**A**–**C**) The morphology of IVP blastocysts. Original magnification: ×100. (**D**–**F**) The nuclear state of IVP blastocysts, DAPI staining. Original magnification: ×200.

Before fertilization, in vitro-matured oocytes were cultured in the aging medium for 12 h, since this time was sufficient to reduce the developmental capacity of bovine ova [25] and, in our prolonged culture system, 24 h of aging led to an extremely poor yield of blastocysts. Oocyte aging in the control medium did not change the cleavage rate, but decreased the subsequent yield of blastocysts (*p* < 0.001) in both the CEO (10.5%) and DO groups (7.9%) compared to the group of freshly matured CEOs (31.1%) (Figure 9 and Figure 10).

**Figure 11:**
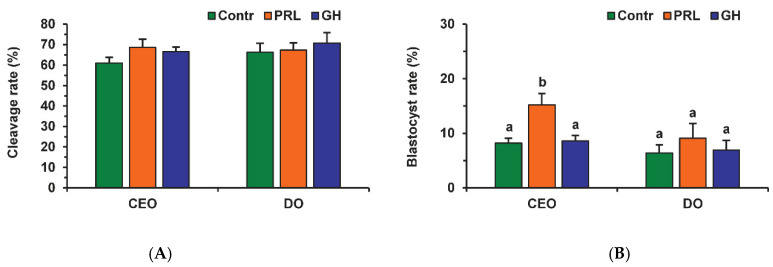
Effects of PRL (50 ng/mL) and GH (10 ng/mL) during the 12 h aging of bovine CEOs and DOs on the oocyte developmental capacity: (**A**) cleavage rate, (**B**) blastocyst rate. Data represent means ± SEM of 6 replicates using 173–184 oocytes per treatment. Means marked with different letters differ significantly (at least *p* < 0.05).

**Figure 12:**
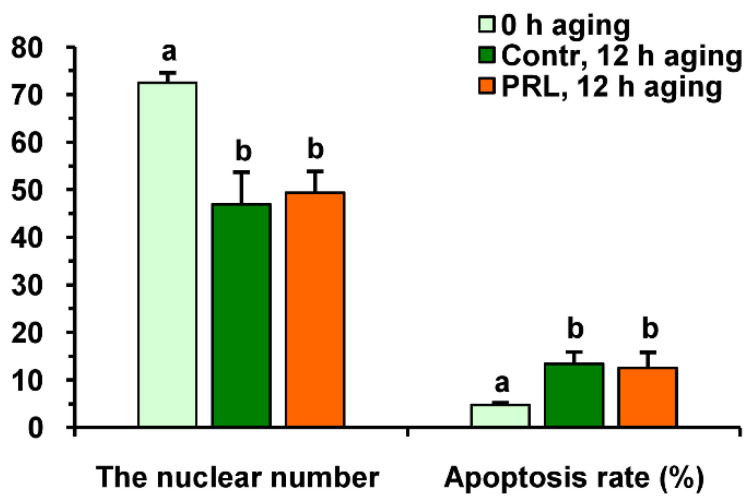
The quality of embryos derived from in vitro-matured bovine CEOs aged for 12 h in the absence or in the presence of PRL (50 ng/mL). Data represent means ± SEM for 46 (0 h of aging), 15 (control, 12 h of aging) and 31 (PRL, 12 h of aging) blastocysts on day 7 after IVF. Means marked with different letters differ significantly (at least *p* < 0.05).

Neither PRL nor GH affected the cleavage rates of aging CEOs and DOs (Figure 11A). However, the supplementation of the aging medium with PRL (but not with GH) caused the blastocyst yield to rise from 8.2% (Control) to 15.2% (*p* < 0.05) (Figure 10 and Figure 11B). When removing cumulus cells, the beneficial effect of PRL was abolished, which reduced the yield to 9.1% (*p* < 0.05). Concurrently, aging of CEOs for 12 h led to a 1.5-fold decrease in the total cell number (Figure 10 and Figure 12) and an almost 3-fold increase in the rate of cell apoptosis in the blastocysts on day 7 (Figure 12 and Figure S4), which is similar to what other researchers had observed [25]. No impact of PRL on the quality of the blastocysts derived from aged ova was found (Figure 12).

**Figure 13:**
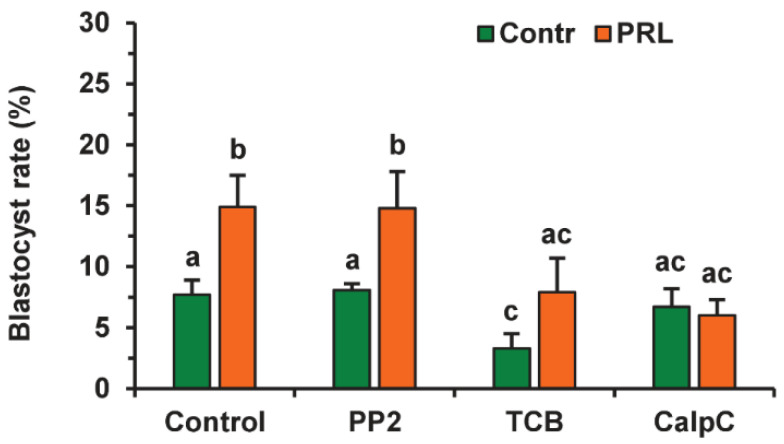
Effects of PRL (50 ng/mL) during the 12 h aging of bovine CEOs in the presence and in the absence of PP2 (10 μM), triciribine (TCB, 25 μM), and calphostin C (CalpC; 0.5 µM) on the subsequent blastocyst formation. Data represent means ± SEM of 6–7 replicates using 177–212 oocytes per treatment. Means marked with different letters differ significantly (at least *p* < 0.05).

Since the beneficial effect of PRL on the developmental capacity of aging oocytes was cumulus-dependent, we tested the involvement of the same signaling pathways as in the case of apoptosis. To avoid a possible adverse influence of the inhibitors used on the oocyte ability for embryonic development, the applied concentrations were two times lower than those used for oocyte apoptosis. None of the inhibitors altered the cleavage rate of CEOs aging in the presence or absence of PRL (Figure S5). Calphostin C (0.5 µM) eliminated (*p* < 0.01) the stimulatory effect of PRL on the blastocyst yield, while it did not affect this yield in the control medium (Figure 13). Triciribine (25 µM) reduced the blastocysts in the control and PRL-treated groups (to 3.3% and 7.9%, respectively, *p* < 0.05). At the same time, the inhibitor of the Src-family tyrosine kinases PP2 (10 µM) did not change the blastocyst yield in either group.

## 3. Discussion

The present research aimed to clarify the effects of two related hormones, PRL and GH, on SPA, apoptosis, and the developmental capacity of matured bovine oocytes during aging in vitro. The data obtained demonstrate for the first time that both hormones can directly support the second meiotic arrest, while only PRL is able to suppress the accelerating effect of surrounding cumulus cells on the apoptosis of aging oocytes, thereby preserving the developmental potential of the latter.

Consistent with our previous findings as well as data from other researchers [24,35], the current study has indicated that SPA is not an early manifestation of oocyte aging in cattle. However, the prolonged culturing of bovine oocytes can be used as a helpful model for the investigation of factors and mechanisms regulating human oocyte spontaneous activation, since the latter is considered one of the causes of multipronucleus formation and aneuploidy cases after intracytoplasmic sperm injection (ICSI) [7]. Our research has shown the cumulus-independent inhibitory effects of PRL and GH on the SPA of bovine ova. The presence of PRL and GH receptors in immature bovine oocytes has previously been revealed [33,34]. Since the oocyte expression of various proteins, including receptors, may be dramatically reduced during oocyte maturation [36], in this study, the expression of PRL and GH receptors in bovine M-II oocytes has been demonstrated, confirming the availability of the direct pathways of hormone signaling into matured ova.

According to current notions, MPF and MAPK, primarily extracellular sig-nal-regulated kinase 1/2 (ERK1/2), are the main factors regulating oocyte activation [7]. In the present research, the effects of both PRL and GH on bovine oocyte SPA were achieved by activating the signaling cascades dependent on SFKs. In pigs, these kinases have been shown to be located in the oocyte membrane, including the M-II stage, and involved in the regulation of MPF and MAPK activities [31]. To date, it has been established that SFKs are coupled constitutively with PRL and GH receptors, and can control the activation of different signaling cascades, including the RAS/RAF/MEK/ERK MAPK signaling pathway [37,38]. However, the effect of only PRL, and not GH, on bovine oocyte activation was mediated by MEK 1/2, highlighting the differences in signal cascades induced by these two hormones downstream of SFKs. Although it is still unknown which SFKs are associated with receptors of PRL and GH, one can assume that the revealed differences in the hormone signaling are due to differences in SFKs associated with PRL and GH receptors. Indeed, the expression of at least three members of the Src family (Fyn, Yes, and Src) has been found in mammalian oocytes, with two of them (Fyn and Yes) involved in the regulation of M-II arrest [39]. One of the possible pathways of GH’s action on oocytes via SFK may be the activation of p38 MAPK [38], another member of the MAPK subfamily, which may also be involved in the regulation of meiotic arrest at the M-II stage [40]. Furthermore, other signaling pathways, such as the participation of SFK, associated with GH receptors, in the control of the MPF activity via inducing cyclin B degradation [31], must not be ruled out. Thus, despite the close relationship of PRL and GH, as well as their receptors, the similar effects of the hormones on bovine oocyte SPA appear to be attained through different effector pathways. Experiments aimed at identifying specific SFKs associated with PRL and GH receptors are required in order to better understand the differences in the actions of the respective hormones on aging oocytes, as well as the general mechanisms supporting M-II arrest.

In the present study, during the prolonged culturing of mature bovine CEOs, the rate of apoptotic oocytes increased in the control group, which is in line with the results of other researchers [25], while PRL suppressed this increase. The removal of cumulus cells also led to a reduction in the frequency of oocyte apoptosis, and abolished the effect of PRL. The findings indicate cumulus’ contribution to the apoptosis of aging oocytes, which is consistent with the results of other investigators [18]. Cumulus cells surrounding aging mouse oocytes have been shown to suffer apoptosis and, in turn, to begin to produce different apoptosis inducers, such as soluble Fas ligand (sFasL) and soluble tumor necrosis factor-α (sTNF-α), which act through their respective receptors in oocytes, as well as ceramide, which is transported to oocytes via gap junctions [17,41]. Taken together, our data and the data of other researchers suggest that the antiapoptotic effect of PRL is due to the elimination of the accelerating effect of senescent cumulus cells on oocyte apoptosis. Here, PRL clearly only exerts its effect during oocyte aging. This suggestion is supported by our earlier findings that the presence of PRL in the medium during the second phase of in the vitro maturation of bovine CEOs does not affect their apoptosis resistance during the subsequent aging [42].

Several possible pathways of the inhibitory effect of PRL on oocyte apoptosis can be suggested: decelerating cumulus cell apoptosis, suppressing the cumulus production of apoptosis inducers, and attenuating the negative action of apoptosis inducers on oocytes. We previously found an inhibitory effect of PRL, mediated by protein kinase C, on the apoptotic degeneration of cumulus cells during the in vitro maturation of bovine CEOs, as well as the presence of PRL receptors in cumulus cells after oocyte maturation [26,43]. However, GH is also able to suppress the apoptosis of the bovine cumulus cells surrounding the maturing oocytes [44], but it did not affect the apoptosis of aging oocytes in the current work. The available data indicate the absence or upregulating effect of PRL on the production of FasL in ovarian cells [45,46], which does not support the assumption that hormonal action reduces the cumulus secretion of FasL. At the same time, it is impossible to exclude the ability of PRL to inhibit the secretion of TNF-α by cumulus cells, similar to its effect on cultured explants of the human placenta [47]. In addition, PRL is able to suppress the apoptosis of human granulosa cells induced by the sphingolipid ceramide [48], which is an important mediator of the programmed death of various cell types, including oocytes [49].

It has been shown that, in human breast cancer cells, PRL can activate sphingo-sine kinase-1 (SK-1) involved in apoptosis downregulation by catalyzing formation of sphingosine 1-phosphate (S1P) counteracting effects of ceramide [49,50]. This action of PRL is attained by the activation of signal transducers and activators of transcription 5 (STAT5), protein kinase C, and MEK/ERK MAPK signaling pathways. PRL is known to transmit its signal into cells through two tyrosine kinases associated constitutively with the PRL receptor, Janus kinase 2 (JAK2) and SFK, with JAK2/STAT5 being the main signaling pathway of the hormone [37]. In the present study, SFKs were not involved in the antiapoptotic effect of PRL on aging bovine oocytes, suggesting that this was implemented via the JAK2/STAT5 pathway. We have also shown that this effect of PRL is mediated by protein kinase C, although it remains unclear whether the activation of protein kinase C occurs in oocytes or cumulus cells, or in both. Furthermore, PRL but not GH was able to activate the MEK/ERK MAPK signaling pathway in the oocytes, protecting them from SPA. Taking into account all these data, it is reasonable to assume that the inhibitory action of PRL on the apoptosis of aging oocytes may be associated with the suppression of the proapoptotic effect of ceramide, which is produced by senescent cumulus cells, through the stimulation of SK-1 in the ova. However, the downregulating effect of PRL on the cumulus-derived TNF-α cannot be completely excluded.

It should be emphasized that the antiapoptotic effect of PRL on aging bovine CEOs was different from its retarding effect on age-related alterations in M-II chromosomes, which we revealed earlier [26], although in both cases the hormone suppressed the adverse influence of cumulus cells. Firstly, the aging-retarding effect of PRL on chromosomes, in addition to protein kinase C, involved SFK and Akt kinase, while its antiapoptotic effect did not depend on both kinases. Secondly, not only PRL, but also GH decelerated the abnormal chromosome changes in the oocytes. This effect of GH might be due to its inhibiting of TNF-α protein expression in cumulus cells, similarly to the hormone’s action on the hypoxic developing mouse brain [51]. This assumption is supported by evidence for the adverse effect of TNF-α on chromosome morphology in aging mouse oocytes [17]. Therefore, it is more likely that the TNF-α pathway is involved in the cumulus-dependent effect of PRL on M-II chromosomes rather than on oocyte apoptosis. Further experiments need to be performed to elucidate the pathways of the antiapoptotic action of PRL on aging oocytes, and particularly the role of the ceramide-related pathway.

Apoptosis is one of the main pathways leading to the poor embryo development of postovulatory aged mammalian oocytes [2]. Accordingly, in the current research, PRL simultaneously suppressed apoptosis in aging bovine oocytes and maintained their developmental ability, with both effects being dependent on the presence of cumulus cells and being mediated by protein kinase C. These data suggest that the beneficial impact of the hormone on the blastocyst yield could be due to its antiapoptotic impact on senescent oocytes. At the same time, despite the fact that cumulus cells accelerated apoptosis, their removal did not enhance the developmental competence of aging oocytes, which was consistent with the vital role of cumulus cells in oocyte functionality [18]. Thus, the use of PRL in the aging medium helped to eliminate the adverse influence of senescent cumulus cells on the quality of oocytes, and thereby supported their ability to develop, although it did not affect the embryo quality further. It is necessary to emphasize that the molecular processes associated with aging are initiated much earlier than morphological and functional changes [1]; therefore, the influence of PRL at the molecular level should also be implemented at a very early period. The antiapoptotic and development-promoting effects of PRL on aging oocytes, together with its suppressive effect on oocyte SPA, point to the possible usefulness of its application in human and animal ART, and primarily in rescue ICSI. According to modern protocols, early rescue ICSI can be performed 4–6 h after insemination, i.e., 8–10 h after ovum pick-up [6], which is sufficient for the initiation of aging processes. Furthermore, there is a physiological rationale for the application of PRL. After ovulation, follicular fluid containing 20–30 ng/mL of PRL flows into the oviduct, in addition to the blood-derived intraoviductal hormone [52,53], allowing prolactin to affect the aging of oocytes.

Apart from the antiaging effects of PRL, a controversial role of Akt kinase in oocyte aging should be noted. In mice, a reduction in the expression of Akt in aging oocytes and an increase in its expression in the surrounding cumulus cells have been demonstrated [15,54]. In the present study, the inhibitor of the Akt kinase triciribine decelerated the aging of DOs, decreasing their SPA, and accelerated the aging of CEOs, increasing their apoptosis rate and reducing the blastocyst yield. Previously, we also found that triciribine suppresses the cumulus-mediated retarding effect of PRL on abnormal chromosome changes in aging bovine oocytes [26]. Therefore, it can be speculated that the decrease in Akt expression in oocytes and its increase in cumulus cells reflect the adaptive ability of CEOs to maintain their quality during aging.

## 4. Materials and Methods

Unless otherwise stated, all media and chemicals were purchased from Sigma-Aldrich Chemical Co. (St. Louis, MO, USA).

### 4.1. Isolation of Oocytes and Conditions of In Vitro Maturation (IVM)

Ovaries of cows and heifers obtained at a slaughterhouse were delivered to the laboratory in sterile saline at 30–35 °C within 3 h of slaughter. The isolation of CEOs was carried out by dissection of the wall of antral follicles 2–8 mm in diameter. All manipulations with oocytes were performed on a heating table at 37 °C. The CEOs were washed several times in HEPES-buffered TCM-199 supplemented with 5% fetal calf serum (FCS; Hyclone Laboratories, Logan, UT, USA), 10 μg/mL of heparin, 0.2 mM sodium pyruvate, and 50 μg/mL of gentamycin sulfate, and then evaluated under a stereomicroscope. Oocytes with fine-grained homogeneous ooplasm surrounded by a compact multilayered cumulus were selected for research. Thereafter, groups of 25–35 CEOs were transferred to 500 μL of a maturation medium (TCM-199 containing 0.2 mM sodium pyruvate, 50 μg/mL of gentamycin, 10% FCS, 10 μg/mL of ovine luteinizing hormone, and 10 μg/mL of porcine follicle-stimulating hormone) overlaid with 500 μL of mineral oil. Oocytes were cultured for 20 h at 38.5 °C in humidified air containing 5% CO_2_.

### 4.2. Prolonged Culture of Aging Oocytes

Some of the matured CEOs were instantly transferred to an aging medium (TCM-199 supplemented with 0.2 mM sodium pyruvate, 50 μg/mL of gentamycin, and 10% FCS) for prolonged culture. Another part of the oocytes was freed from cumulus cells by treatment with 0.1% hyaluronidase in the aging medium, as described previously [26]. The quality of cumulus removal was monitored using an inverted light microscope (at magnification ×200). After several washes, the DOs were subjected to prolonged culture. The oocytes were cultured in the aging medium in the absence (control) and presence of 50 ng/mL of PRL (USDA bPRL B-1, Beltsville, MD, USA) or 10 ng/mL of GH (Monsanto, St. Louis, MO, USA) under the above conditions. Frozen aliquots of a stock solution (50 μg/mL of PRL or 10 μg/mL of GH in saline) were diluted by the aging medium immediately prior to culture.

In the first experiment, CEOs and DOs were incubated for 0, 12, 24, 36, or 48 h with and without PRL or GH. In addition, DOs were cultured for 44 h in the absence and in the presence of either PRL or GH and/or inhibitors of different signaling pathways. The following inhibitors were applied: (1) PP2, the inhibitor of Src-family tyrosine kinases (20 μM), (2) triciribine, the inhibitor of Akt kinase (50 μM), (3) U0126, the MEK 1/2 inhibitor (20 μM; Promega, Madison, WI, USA), and (4) L-NAME, the efficient inhibitor of both eNOS and nNOS (20 μM). At the end of culture, the rate of oocyte SPA was assessed.

At the first step of the second experiment, CEOs and DOs were incubated for 0 h and 24 h with and without PRL or GH. At the second step, CEOs were cultured for 24 h in the absence and in the presence of PRL and/or protein kinase inhibitors. The following inhibitors were used: (1) PP2 (20 μM), (2) triciribine (50 μM), and (3) calphostin C, the protein kinase C inhibitor (1 μM; Calbiochem, Darmstadt, Germany). Following culture, the yield of apoptotic oocytes was determined.

In the third experiment, CEOs and DOs were cultured for 0 h and 12 h with and without PRL or GH. Furthermore, CEOs were cultured for 12 h in the absence and in the presence of PRL and/or PP2 (10 μM), triciribine (25 μM), or calphostin C (0.5 μM). After culture, the oocytes underwent in vitro fertilization.

### 4.3. Assessment of Oocyte Activation

To assess the SPA of aged bovine oocytes, cytogenetic preparations were performed via the method of Tarkowski [55] with some modifications [56]. The state of the nuclear material in oocytes and presumable parthenotes was examined under a light microscope (Axio Scope.A1; Carl Zeiss, Oberkochen, Germany) at magnification ×1000 using criteria described earlier [57]. The number of oocytes that underwent SPA was determined by summarizing the numbers of parthenogenetic embryos cleaved and the oocytes that reached anaphase-II to pronucleus stages. The rate of activated oocytes was expressed as a percentage of the total number of M-II oocytes.

### 4.4. Immunofluorescent and Immunocytochemical Detection of Receptors

The protein expression of PRL and GH receptors in oocytes matured in vitro was detected by immunofluorescence and immunocytochemical staining. Unless otherwise specified, all procedures were performed at room temperature. Immediately after IVM, the oocytes were freed from cumulus cells as described above. Following washing with PBS containing 0.2% BSA, the oocytes were fixed for 20 min with 4% paraformaldehyde in PBS. The permeabilizing treatment of specimens and the blocking of nonspecific binding were performed in accordance with procedures reported previously [26]. Thereafter, the oocytes were incubated with primary antibodies against PRL or GH receptor, mouse monoclonal antibody MA1-610 (Thermo Scientific, Rockford, IL, USA; 1:50 dilution) or mouse monoclonal antibody MAB 263 (Abcam, Cambridge, MA, USA; 1:40 dilution), for 16 h at 4 °C.

For immunofluorescence, the oocytes were incubated with Alexa fluor 488-labeled goat anti-mouse IgG (Thermo Fisher Scientific, Waltham, MA, USA; 1:50 dilution) for 2 h in the dark. All antibodies were diluted in PBS containing 1% BSA and 3% goat serum. After DNA staining with 4′,6′-diamidino-2-phenylindole solution (DAPI; 1 μg/mL in PBS) for 20 min, the oocytes were washed several times in PBS–BSA and mounted on superfrost slides (Thermo Scientific) in the antifade medium Vectashield (Vector Laboratories, Inc., Burlingame, CA, USA). All specimens were evaluated for the presence of PRL and GH receptors using a fluorescent microscope (Axio Imager.M2; Carl Zeiss, Oberkochen, Germany) at magnification ×400.

In the case of immunocytochemistry, the oocytes were incubated with biotinylated goat anti-mouse IgG (Vector Laboratories, Burlingame, CA, USA; 1:500 dilution) and then with Vectastain ABC reagent and 3-amino-9-ethylcarbazole (AEC) substrate (Vector Laboratories), as described earlier [26]. The samples were counterstained with hematoxylin and mounted in the glycerol-PBS mixture (1:3). All preparations were examined under a light microscope at magnification ×400.

No staining was revealed in the negative controls, derived by omitting the primary antibodies. Total of 19 oocytes and 25 oocytes were used for the immunodetection of PRL and GH receptors, respectively.

### 4.5. In Vitro Fertilization (IVF) and Embryo Culture (IVC)

Immediately after in vitro maturation or 12 h aging, the CEOs and DOs were washed once in Fert-TALP medium (114 mM NaCl, 3.2 mM KCl, 0.4 mM NaH_2_PO_4_·2H_2_O, 2 mM CaCl_2_·2H_2_O, 0.5 mM MgCl_2_·6H_2_O, 25 mM NaHCO_3_, 10 mM HEPES, 10 mM sodium lactate, 0.25 mM sodium pyruvate, 6 mg/mL of BSA, 0.1% non-essential amino acids, and 50 μg/mL of gentamycin) supplemented with 10 μg/mL of heparin, 20 μM penicillamine, 10 μM hypotaurine, and 1 μM epinephrine, and transferred to four-well dishes (Nunc, Roskilde, Denmark) containing 400 µL of the same medium overlaid with equal volumes of mineral oil. The fertilization of the oocytes was conducted using frozen/thawed semen from one bull of proven fertility. To this end, 1.5 h before oocyte fertilization, straws with frozen semen were thawed and active sperms were obtained by the swim-up method [58] with the use of Sperm-TALP medium (100 mM NaCl, 3.1 mM KC1, 0.3 mM NaH_2_PO_4_·2H_2_O, 2 mM CaCl_2_·2H_2_O, 1.5 mM MgCl_2_·6H_2_O, 25 mM NaHCO_3_, 10 mM HEPES, 21.6 mM sodium lactate, 1 mM sodium pyruvate, 6 mg/mL of BSA, and 50 μg/mL of gentamycin). The contents of the straws (220 μL) were layered under 1 mL of Sperm-TALP medium in 1.8 mL tubes (Nunc, Denmark) and placed in a CO_2_ incubator (MCO-18AIC, Sanyo, Moriguchi, Japan) for 50 min. At the end of incubation, 750 μL of the upper layer was taken from the tubes, followed by dilution with fresh Sperm-TALP medium and centrifugation at 300× *g* for 7 min. The resulting pellet, comprising motile sperm, was added to the Fert-TALP medium containing matured oocytes to a final concentration of 1.5 × 10^6^ spermatozoa per mL. Oocytes were co-incubated with sperm for 16–18 h at 38.5 °C under 5% CO_2_ in humidified air.

After co-incubation with sperm, the oocytes were carefully pipetted and washed in Fert-TALP medium for the deleting of cumulus cells and adhered spermatozoa. Putative zygotes were transferred to the four-well dishes containing 500 µL of CR1 medium supplemented with essential and nonessential amino acids (CR1aa) [59] coated with equal volumes of mineral oil and cultured for 4 days at 38.5 °C under 5% CO_2_ in humidified air. Thereupon, the cleaved embryos were placed to the fresh CR1aa medium supplemented with 5% FCS and cultured for 3 days under the same conditions. Embryo development was assessed under a stereomicroscope (SMZ, Nikon, Minato, Tokyo, Japan) at days 2 and 7 post-insemination for cleavage and blastocyst formation.

### 4.6. Terminal Deoxynucleotidyl Transferase-Mediated 2′-Deoxyuridine 5′-Triphosphate (dUTP) Nick-End Labeling (TUNEL) Assay and DAPI Staining

Apoptosis was detected in bovine oocytes and embryos using the In Situ Cell Death Detection Kit, Fluorescein (Roche Diagnostics, Mannheim, Germany) [60,61]. Briefly, the matured or aged oocytes denuded of their cumulus cells and the day 7 blastocysts were fixed with 4% paraformaldehyde in PBS for 1 h and then permeabilized with 0.5% Triton X-100 in 0.1% sodium citrate for 1 h at room temperature. Positive controls were treated with 50 U/mL DNase I recombinant (Roche Diagnostics) at 37 °C for 1 h. Experimental oocytes and embryos as well as positive controls were incubated in the TUNEL reaction mixture consisting of fluorescein-conjugated dUTP and terminal deoxynucleotidyl transferase for 1 h at 37 °C in the dark. Negative controls were incubated under the same conditions with labeled dUTP in the absence of terminal deoxynucleotidyl transferase. All specimens were counterstained with DAPI, placed in the antifade medium, and assessed under the fluorescent microscope as described above. Meiotic stages were determined via the DAPI staining (blue) and apoptotic oocytes were detected by the TUNEL-positive staining (green) of chromosomes. The yield of apoptotic oocytes was expressed as a percentage of the total number of M-II oocytes. In blastocysts, the ratio of TUNEL-positive and DAPI-labeled nuclei expressed in percent was taken as the apoptosis rate.

### 4.7. Statistical Analysis

Results were obtained from 4–7 independent experiments and expressed as means ± SEM. The number of oocytes in each experimental group is indicated in the figure captions. Statistical differences were assessed by one-way or two-way ANOVA followed by the Tukey’s HSD test with SigmaStat 4.0 software (Systat Software, Inc., San Jose, CA, USA). The data were subjected to arcsine transformation in the absence of normal distribution or homogeneity of variance. When using two-way ANOVA, treatments with PRL or GH and an aging period, and treatments with inhibitors, were the independent variables. Differences were considered statistically significant with a probability of *p* < 0.05.

## 5. Conclusions

In general, the findings of the current research indicate for the first time that PRL and GH can directly support meiotic arrest in aging bovine M-II oocytes by activating MAP kinases and/or Src-family kinases. By contrast, only PRL is able to attenuate the adverse effect of senescent cumulus cells on the apoptotic degeneration of oocytes, and thereby to maintain their ability to develop. Meanwhile, both the antiapoptotic and development-promoting effects of PRL on aging bovine oocytes are related to the pro-survival action of the protein kinase C-mediated signal pathway. The data propose the suitability of using PRL as an antiaging agent in human and animal ART. Further experiments are needed to clarify the mechanisms of the inhibitory effects of PRL on oocyte SPA and the apoptosis-accelerating actions of cumulus cells.

## Data Availability

The data presented in this research are available in the article and Supplementary Materials.

## Supplementary Materials

The following are available online at https://www.mdpi.com/article/10.3390/ph14070684/s1, Figure S1: Morphology of the nuclear material at various stages of spontaneous parthenogenetic activation of aged bovine oocytes (cytogenetic preparations), Figure S2: Immunocytochemical detection of PRL and GH receptors in M-II oocytes using a brown DAB-chromophore, Figure S3: Representative images of apoptosis detection in matured bovine oocytes by TUNEL assay, Figure S4: Representative images illustrating detection of apoptosis in bovine day 7 embryos, Figure S5: Effects of PRL (50 ng/mL) during the 12 h aging of bovine CEOs in the presence or absence of PP2 (10 μM), triciribine (50 μM), and calphostin C (0.5 µM) on subsequent embryo cleavage.
