# Supplementary material for: Delaying Effects of Prolactin and Growth Hormone on Aging Processes in Bovine Oocytes Matured In Vitro
Source: Pharmaceuticals (Basel). 2021 Jul 16;14(7):684. doi: 10.3390/ph14070684 (PMC8308928; doi:10.3390/ph14070684)
Supplement: Supplementary file 1 [file pharmaceuticals-14-00684-s001.zip › pharmaceuticals-1273420-supplementary.pdf]

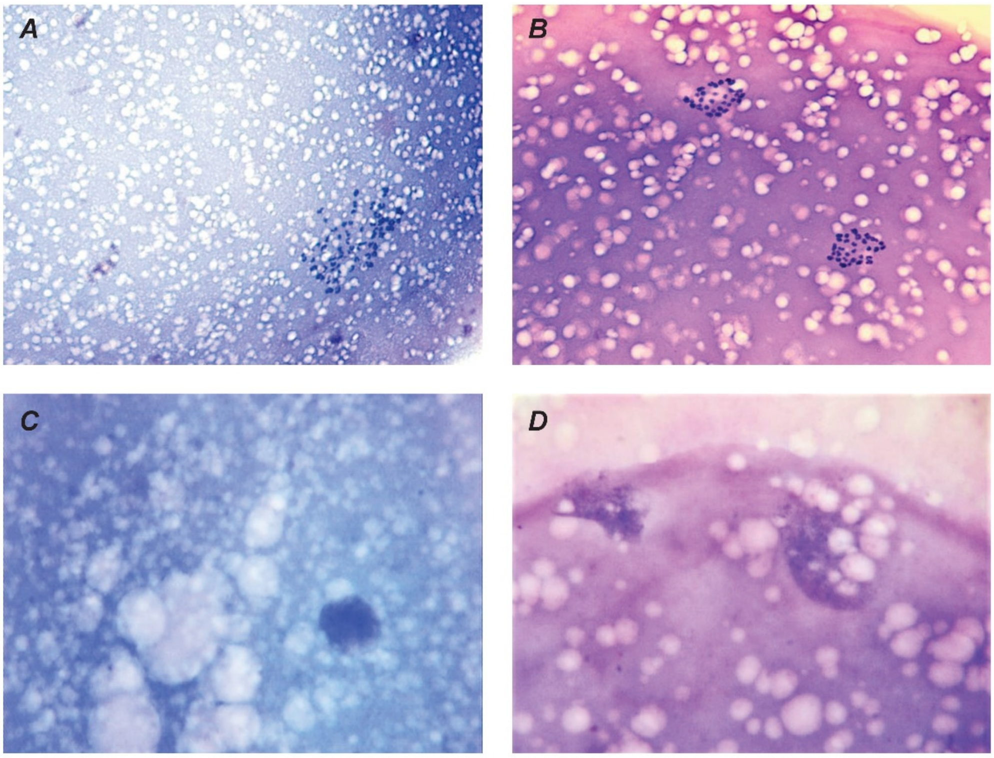

Figure S1. Morphology of the nuclear material at various stages of spontaneous parthenogenetic activation of aged bovine oocytes (cytogenetic preparations): (**A**) anaphase-II, (**B**) telophase-II, (**C**) pronucleus stage, (**D**) two-cell embryo. Original magnification: × 1000.

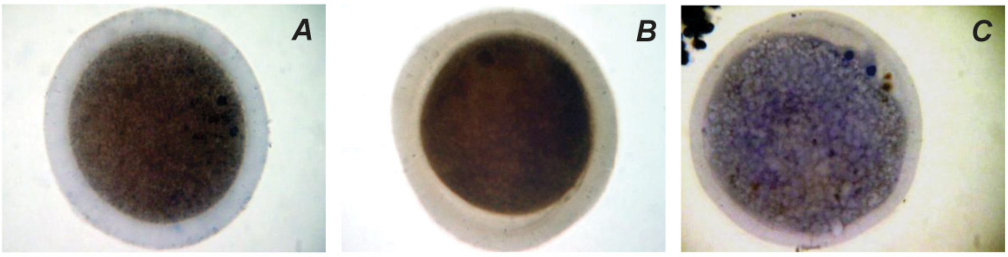

Figure S2. Immunocytochemical detection of PRL and GH receptors in M-II oocytes using a brown DAB-chromophore. (**A**) Positive staining for PRL receptors with MA1-610 antibody. (**B**) Positive staining for GH receptors with MAB 263 antibody. (**C**) Negative control performed by omitting primary antibodies. Nuclei were counterstained with hematoxylin. Original magnification: × 400.

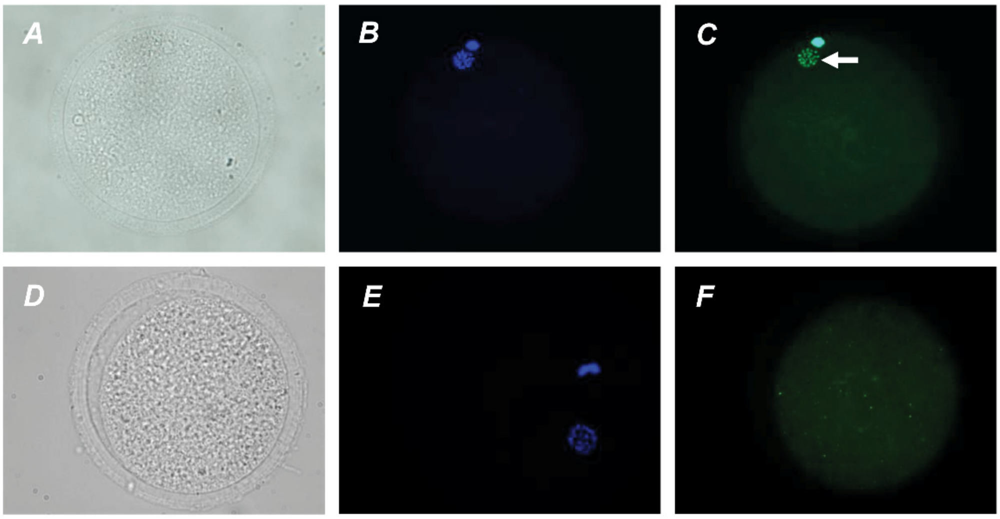

Figure S3. Representative images of apoptosis detection in matured bovine oocytes by TUNEL assay. (**A, D**) Bright-field images. (**B, E**) DNA staining with DAPI (blue). (**C**) TUNEL-positive staining (green) of chromosomes is indicated by a white arrow. (**F**) TUNEL-negative staining. Original magnification: × 400.

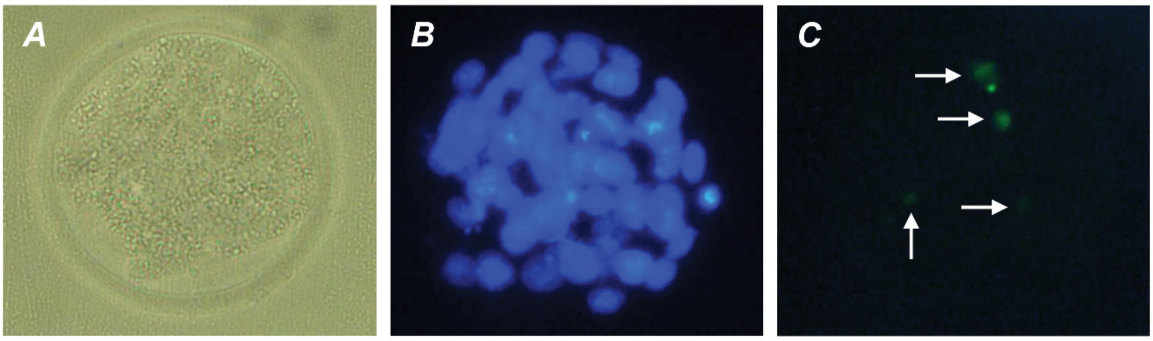

Figure S4. Representative images illustrating detection of apoptosis in bovine day 7 embryos. (**A**) bright-field images. (**B**) DAPI staining of nuclei (blue). (**C**) TUNEL staining of nuclei (green). TUNEL-positive nuclei are indicated by white arrows. Original magnification: × 200.

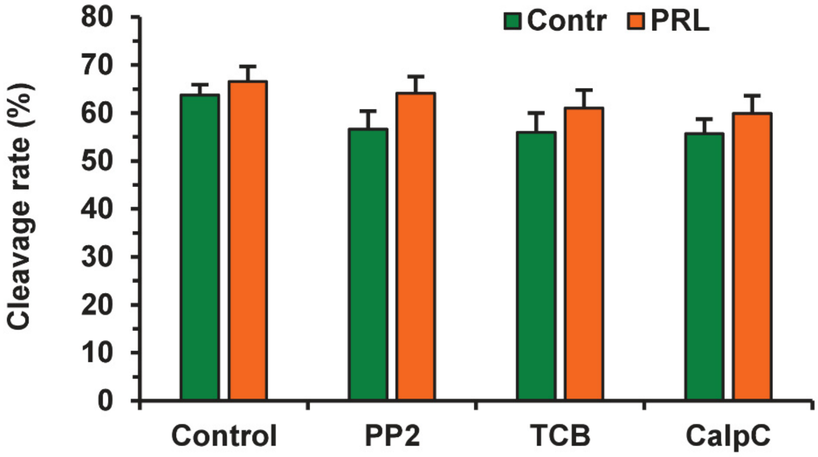

Figure S5. Effects of PRL (50 ng/ml) during the 12 h aging of bovine CEOs in the presence or absence of PP2 (10 μM), triciribine (50 μM), and calphostin C (0.5 μM) on subsequent embryo cleavage. Data represent means ± SEM of 6-7 replicates using 177–212 oocytes per treatment. Means with different letters differ significantly (at least $p < 0.05$).
